# Novel and Emerging Tools and Technologies in Cardiac Electrophysiology: What’s on the Horizon in 2020?

**DOI:** 10.19102/icrm.2019.101206

**Published:** 2019-12-15

**Authors:** Arash Aryana

**Affiliations:** ^1^Mercy General Hospital and Dignity Health Heart and Vascular Institute, Sacramento, CA, USA

**Keywords:** Cardiac mapping, catheter ablation, fluoroscopy, radiofrequency

During the last decade, the field of invasive cardiac electrophysiology has experienced significant and steady progress, largely through the refinement of existing tools and catheters. For instance, the introduction of high-density electroanatomic mapping (EAM); force-sensing ablation catheters; and the adjustment/modification of ablation parameters such as power, duration, and irrigation have led to progressive improvements in mapping and ablation safety and efficacy as well as greater procedural efficiency (ie, shorter durations) and reductions in fluoroscopic need/exposure. Still, the best may yet be to come: it appears that the upcoming decade will likely bring into focus novel mapping and ablation tools, strategies, and even energy modalities, all offering enormous potential to further transform the field. Below, some of these promising tools and technologies are briefly examined.

## Emerging mapping and navigation technologies

The introduction of three-dimensional (3D) EAM systems has facilitated marked improvements in catheter visualization, arrhythmia mapping, and the reduction and even elimination of fluoroscopy/radiation exposure in cardiac electrophysiology.^1^ Presently, the most commonly used EAM systems include CARTO^®^ (Biosense Webster, Diamond Bar, CA, USA), EnSite™ NavX™ (Abbott Laboratories, Chicago, IL, USA), and Rhythmia HDx™ (Boston Scientific, Natick, MA, USA). The former uses an electromagnetic field created with six patches placed on the patient’s chest and back. Consequently, a large number of locations within the cardiac chamber(s) are recorded by the system during intracardiac movement of proprietary mapping/ablation catheters to create a 3D EAM.^2^ On the other hand, the EnSite NavX system (Abbott Laboratories, Chicago, IL, USA) is based on an electric field created using three pairs of orthogonal patches along the x-, y- and z-axes located on the patient’s chest and abdomen serving as references, whereas the latter system (Rhythmia HDx™; Boston Scientific, Natick, MA, USA) relies on both magnetic- and impedance-guided catheter localization.^2^

Meanwhile, newer mapping systems have recently been launched that have the potential to further reduce the need for fluoroscopy. The recently introduced AcQMap system (Acutus Medical, Carlsbad, CA, USA) combines ultrasound-guided anatomical views with bipolar/unipolar signals obtained with the application of a diagnostic catheter to create high-resolution 3D EAMs.^3^ The diagnostic recording catheter consists of a hybrid spheroid with six splines, each populated with eight ultrasound transducers and eight biopotential electrodes for a total of 48 ultrasonic transducers and 48 electrodes. The system can sample the cardiac chamber at a rate of 115,000 points/min. Of great interest is this system’s ability to track catheter movement in real time when combined with a magnetic resonance map of the heart.^4^

**Figure 1:**
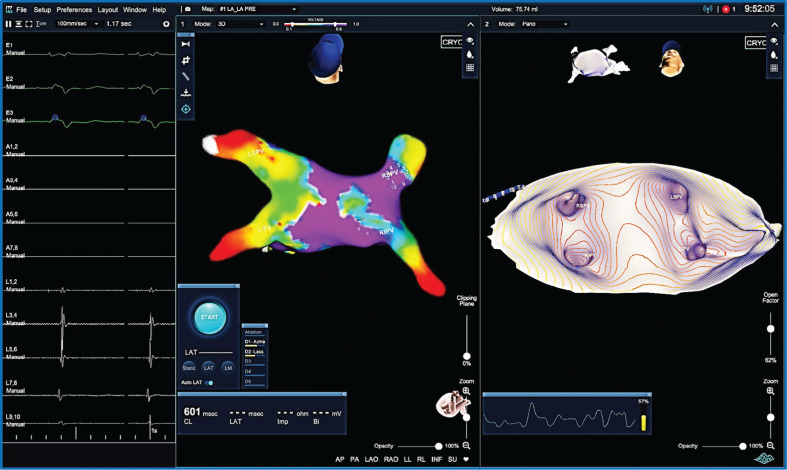
The KODEX cardiac 3D imaging and navigation system (Phillips Healthcare, Amsterdam, the Netherlands). Shown are **A:** a 3D anatomical reconstruction and **B:** a panoramic view depicting the left atrial and pulmonary venous anatomy in a patient undergoing catheter ablation of atrial fibrillation. Images courtesy of Darryl Wells, MD.

KODEX-EPD (Philips Healthcare, Amsterdam, the Netherlands) is another novel, nonfluoroscopic, dielectric-based imaging and navigation system that enables 3D real-time visualization of intracardiac catheter placement through shifting voltage and electric field measurements, as electrode-containing catheters move within an electric field produced by disposable body surface patches **(Figure 1)**. Specifically, this system measures the electromagnetic properties of tissue and uses internal and external electrodes to transmit and receive low-amplitude frequencies and phased electrical signals. The data are then utilized to derive an electromagnetic signature for the material between the transmitting and the receiving ports, which is, in turn, used to determine location as well as contact pressure. The system’s spatial resolution—namely, the capability to distinguish between two closely-positioned points within an ablation area, is approximately 0.3 mm.^5^ Consequently, this system generates high-resolution 3D cardiac anatomical images without the need for direct catheter–tissue contact or preacquired computed tomography/magnetic resonance imaging. Unlike some of the other mapping systems that employ hybrid localization and vendor-specific catheters, this technology could emerge as the ultimate open-platform system and operate with virtually any intracardiac mapping/ablation catheter/electrode technology.^6,7^ If successful, this system also has the potential to markedly facilitate procedures such as cryoballoon ablation of atrial fibrillation and perhaps even the implantation of cardiovascular implantable electronic devices through real-time and/or panoramic visualization as well as force-sensing, which are currently not options offered for such procedures, while simultaneously eliminating the need for fluoroscopy. Additionally, the system’s ability to assess tissue changes could aid in the validation of lesion formation and the identification of ablation gaps. Although this system’s accuracy has been compared to that of currently available EAM systems,^5^ further research is much needed to elucidate this system’s reliability and precise role and potential in cardiac electrophysiology.

## Emerging ablation tools and energy modalities

Radiofrequency (RF) delivery using an irrigated 3.5-/4-mm-tip ablation catheter remains the principal mode of ablation for the treatment of common cardiac arrhythmias such as atrial fibrillation. However, alternate ablation catheter configurations including a multielectrode RF balloon (HelioStar; Biosense Webster, Diamond Bar, CA, USA) as well as a novel, expandable spheroid RF ablation catheter (Affera, Watertown, MA, USA) are also under investigation. Recently, the outcomes of catheter ablation using the latter were reported.^8^ This high-current, low-density ablation system is capable of creating “wide and shallow” lesions in the atrial/right ventricular tissue and “large and deep” lesions within the left ventricle in a short duration, seemingly with superb durability and safety and a low risk of steam pops/thrombus.^8,9^ This technology differs from conventional ablation systems and, to fully understand the concept behind its mechanism, it is worthwhile to first review the biophysics of RF ablation themselves.

**Figure 2:**
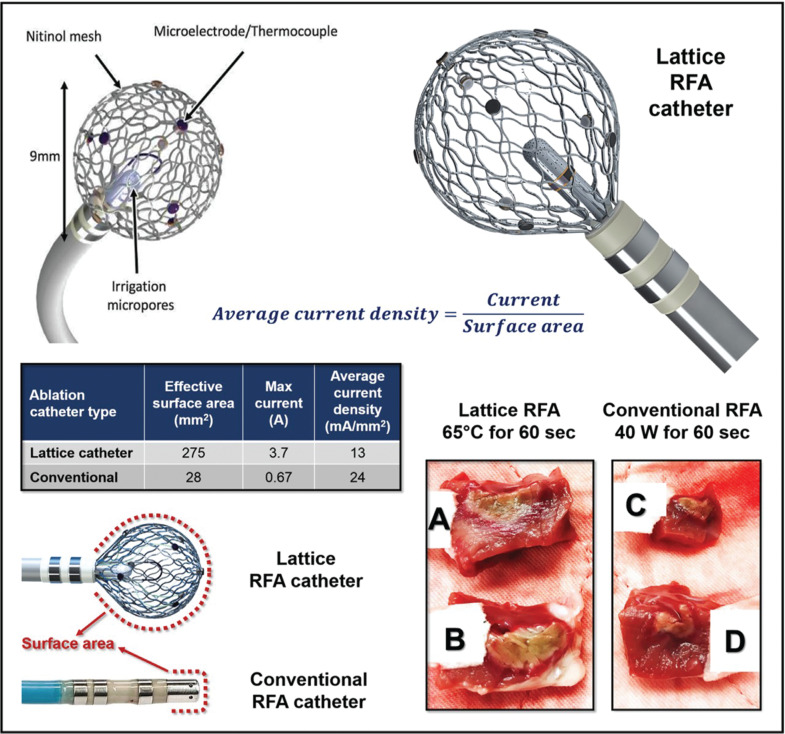
The lattice RF ablation catheter. Shown is the lattice catheter design consisting of a 9-mm, 7.5-French spheroid-shaped bidirectional deflectable ablation catheter with an expandable conductive nitinol mesh that contains nine thermocouple sensors distributed uniformly on its surface. At its center sits an irrigation nozzle with micropores that provide homogeneous irrigation/cooling. Ablation is performed in a temperature-controlled mode using high power. As compared with a conventional irrigated RF ablation catheter, as shown in the table, the lattice catheter design has a surface area that is roughly one order of magnitude greater (275 versus 28 mm^2^). Since the current density is defined as current divided by surface area (ie, the equation), this allows effective delivery of a higher current while maintaining a low current density equal to nearly one-half that of a conventional RF ablation catheter (13 versus 24 A/mm^2^). The lower right panels illustrate a cross-section of ablation lesions created with these ablation parameters using the lattice **(A, B)** versus a conventional irrigated RF ablation catheter **(C, D).** As seen, ablation lesions created using the lattice **(A, B)** are significantly larger in depth and width.

In traditional ablation systems, RF energy is delivered by applying alternating current from the tip of the ablation electrode through a resistive volume of mass composed of myocardial tissue and blood to a patch located on the surface of the patient’s body.^10^ Upon passing the current through the resistive tissue, heat is generated, which raises the tissue temperature, leading to irreversible cellular thermal injury and necrosis.^11^ Hence, there is a direct relationship here between current and temperature such that increased current results in greater tissue heating and larger ablation lesions.^12^ Having said that, this phenomenon is greatly limited by the maximum amount of current that can be safely delivered without creating tissue overheating or steam pops. This threshold is dependent on the current density, which is defined as the ratio of total current to the effective surface area.^13^ To facilitate increased delivery of power while maintaining a low risk for tissue overheating, the current can be delivered over a larger surface area to keep the current density low, and the lattice ablation catheter **(Figure 2)** is designed to meet this objective. It consists of a 7.5-French, 9-mm spheroid-shaped bidirectional deflectable ablation catheter with an expandable conductive nitinol mesh that contains nine temperature sensors distributed uniformly on its surface.^8^ A protected irrigation nozzle is located at its center with micropores that provide homogeneous cooling, reportedly unaffected by the pressure applied on the tissue or the catheter itself. It operates via a high-power RF generator that performs ablation in a temperature-controlled mode. The catheter’s temperature sensors record the rise in temperature during energy delivery in the tissue, as the system provides an indication of tissue contact through impedance measurements.

To date, several investigators^8,9,13–17^ have reported on the ablation outcomes achieved using this novel system. Radiofrequency ablation for only five to seven seconds using this system over thigh muscle and atrial tissue preparations typically yield lesions measuring 10 mm to 15 mm in width and ~4 mm in depth with excellent durability, whereas longer applications (60–120 seconds) on bovine myocardium have proven capable of creating 8-mm- to 10-mm-deep lesions.^8,9^ Preliminary experience with the ablation of the cavotricuspid isthmus and mitral isthmus as well as in pulmonary vein isolation have yielded favorable results, including remarkably short ablation and procedure times and a low risk of steam pops or thrombus formation.^14–17^ Although the striking efficacy and safety outcomes associated with this ablation system appear quite promising, large-scale multicenter clinical trials are still needed to corroborate these early findings. Alternatively, other novel and promising energy modalities are also under investigation. To date, a variety of energy sources have been evaluated for catheter ablation including microwave, high-intensity focused ultrasound, low-intensity collimated ultrasound, laser, cryothermy, and hot saline. Irrespective of the energy source, however, thermal ablation is categorically met with safety challenges commonly related to a lack of control over the extent of ablation lesions that can result in unintended collateral injury. Yet, an energy source that holds great promise is pulsed electric-field ablation.

Pulsed electric fields create tiny defects in the phospholipid bilayer of the cellular outer membrane, thereby increasing its permeability. Neumann et al.^18^ originally published on the results of electric field-mediated DNA uptake into cells that subsequently expressed the foreign gene, coining the term “electroporation.” Reversible electroporation (RE) allows for the transient formation of pores within the cell membrane to promote the uptake of material while also enabling the cell to fully recover. The initial applications in the area of RE involved increasing cell permeability to facilitate gene transfer and the delivery of therapies such as chemotherapeutic drugs into tumor cells (ie, electrochemotherapy). However, the tissue response to an applied electric field depends highly upon the strength and timing of the applied field.

When the field strength is above a critical threshold, this mode of cell permeabilization is known as irreversible electroporation (IRE). The role of IRE to promote nonthermal cardiac ablation is not by any means a new concept. In fact, direct-current shock delivery was utilized by Gallagher et al.^19^ as the original energy source with which they performed early cardiac ablations. Years later, the mechanism of lesion formation in this treatment approach was determined to be IRE.^20,21^ More recently, the IRE of cardiac tissue has been reexamined.^22–27^ Yet, the studied systems vastly differ in regard to device platform, energy source, electroporation parameters, and delivery method. That being said, clearly, there are unique benefits attributed to this novel ablation modality. Aside from an ultra-rapid mode of ablation, electroporation can also be tailored based upon tissue specificity to preserve sensitive structures in the path of ablation energy (eg, vessels, nerves).^28–30^ Furthermore, IRE is unaffected by the “heat-sink” effect that can sometimes impede conventional forms of thermal ablation. Recently, the term “pulsed field ablation” has been used to describe nonthermal monophasic- or biphasic-waveform, field-based cardiac arrhythmia ablation.^27,29–31^ Each therapeutic application consists of a series of electric pulses delivered as a pulse train. For bipolar systems, during each pulse, current flows in a bipolar manner between individual electrodes on the multielectrode catheter, which radiates an electric field that surrounds the catheter. In a unipolar system, the electric field is driven from the catheter to a patch on the patient’s back. After exposure to a series of pulse trains, the hyperpermeabilization of the cell membrane becomes permanent, which leads to cardiomyocyte death followed by lesion formation through replacement fibrosis.^30^ During each pulse, current flows in a bipolar manner between individual electrodes typically on a multielectrode catheter that radiates an electric field to surround the catheter. After exposure to a series of pulse trains, the hyperpermeabilization of the cell membrane becomes permanent, which leads to cell death. Preliminary data suggest that ablation lesions created in the targeted myocardium using this approach are durable, ensuring preserved integrity and function of collateral structures such as the esophagus, lungs, pulmonary veins, coronary arteries, and the vagus and phrenic nerves.^29–31^ The focus of development of this new technology has been to optimize pulse parameters to reduce complications, including char formation and thermally mediated embolic events. While not observed to date, high-voltage applications driving high-level current still have the potential to create heat in the immediate vicinity of the electrode, thereby increasing the probability of char formation and thermally mediated embolic events.^32,33^ Although this has not been observed to date, high-voltage applications driving high-level current still have the potential to create heat in the immediate vicinity of the electrode, thereby increasing the probability of char formation and thermally-mediated embolic events.^22,30^ Further investigation into this technology is clearly warranted, not only regarding its acute and long-term clinical safety and efficacy but also to shed light on the optimal parameters required during ablation such as the pulse amplitude (applied voltage), pulse width, number of pulses delivered in a given pulse train, and number of pulse trains delivered at each application site.

Hence, with the end of 2019 drawing near, it seems that the decade that lies ahead will continue to bring remarkable and innovative changes to the field of cardiac electrophysiology. While some of the improvements will be driven by the progressive evolution of existing tools and products, indeed, there are a handful of novel and potentially disruptive technologies on the horizon as well. Such technological developments will likely advance our abilities in the areas of catheter mapping and ablation of cardiac arrhythmias, which will in turn further enhance patient and procedural outcomes.
